# Engineering high-fidelity tapasin variants to enhance MHC-I antigen presentation

**DOI:** 10.1016/j.jbc.2026.111400

**Published:** 2026-03-21

**Authors:** Molly C. Erdman, Kaya Epstein, Daniel Hwang, Radia M.M. Khan, Shirley M. Sun, Nikolaos G. Sgourakis

**Affiliations:** 1 Center for Computational and Genomic Medicine, Department of Pathology and Laboratory Medicine, The Children’s Hospital of Philadelphia, Philadelphia, Pennsylvania, USA; 2 Cancer Biology Graduate Group, Perelman School of Medicine, University of Pennsylvania, Philadelphia, Pennsylvania, USA; 3 Department of Biochemistry and Biophysics, Perelman School of Medicine, University of Pennsylvania, Philadelphia, Pennsylvania, USA; 4 Bioengineering Graduate Group, University of Pennsylvania, Philadelphia, Pennsylvania, USA

**Keywords:** deep mutational scanning (DMS), antigen processing and presentation, major histocompatibility complex (MHC), protein engineering, high-throughput screening (HTS), functional genomics, neuroblastoma, chaperone

## Abstract

Human leukocyte antigen (HLA) proteins are extremely polymorphic, with different allotypes exhibiting a wide range of dependencies on the chaperone tapasin for peptide loading, expression, and stability at the cell surface. Given its central role in antigen processing, tapasin is frequently downregulated across viral infections and cancers, impairing antigen presentation and hindering the identification of therapeutically relevant peptide antigens. We hypothesized that elucidating the mutational tolerance of tapasin surfaces, which mediate interactions with polymorphic HLA residues, can provide a means for fine-tuning its chaperoning function and reveal mechanistic epitopes that underlie its function. Using two complementary deep mutational scanning screens, we systematically mapped the tapasin/HLA-A∗02:01 interaction landscape, highlighting key regions involved in HLA-I assembly and repertoire optimization. We engineered two high-fidelity tapasin variants, tapasin-YTY and tapasin-I2, and show that these variants can increase cell surface HLA expression on a wild-type (WT) tapasin-deficient background by up to 50% and improve peptide-loading function across multiple HLA-A∗ allotypes. Our findings establish scanning mutagenesis as a general approach for fine-tuning chaperone interactions with HLA molecules to enhance antigen presentation in immunologically impaired settings.

Human leukocyte antigen class I (HLA-I) molecules display a range of processed, intracellular peptides at the cell surface, facilitating immunosurveillance by circulating cytotoxic lymphocytes (1, 2). This selective pressure by the host immune system has resulted in a wide range of immune evasion mechanisms employed by cancerous cells and intracellular pathogens which directly target HLA-I presentation. Amongst these is dysregulation of antigen processing and presentation (APP) machinery (2, 3, 4, 5, 6, 7, 8, 9) (https://www.ncbi.nlm.nih.gov/books/NBK2226/), which reduces the amount of functional peptide-HLA (pHLA) complexes displayed on the cell surface.

The assembly of HLA-I molecules occurs in the endoplasmic reticulum (ER), where association of processed peptides with HLA heavy chain and β_2_-microglobulin (β_2_m) is facilitated by tapasin, a dedicated chaperone and member of the peptide loading complex (1, 2, 10, 11, 12, 13). Tapasin stabilizes empty HLA-I molecules and promotes peptide repertoire optimization by enhancing the exchange of low-affinity for high-affinity peptide ligands to the nascent HLA-I peptide binding groove (1, 11, 12, 14, 15, 16, 17, 18, 19). The essential role of tapasin in promoting HLA-I assembly and repertoire optimization is underscored by its frequent downregulation across human diseases (20, 21, 22, 23). Clinically, tapasin dysregulation is not only linked to worse outcomes across cancers and viral infections but also complicates the identification of therapeutically relevant antigens for immunotherapy design (21, 24). These observations highlight the importance of understanding tapasin-HLA-I interactions, as they may provide key insights into potential mechanisms for overcoming immune evasion and enhancing antigen discovery platforms.

Achieving a molecular understanding of tapasin/HLA-I interactions can be achieved by mapping the mutational tolerance of specific residues through deep mutational scanning (DMS) studies, as we have previously done for the tapasin homolog TAPBPR (25, 26, 27, 28, 29, 30). This allowed us to identify a high-fidelity TAPBPR variant (TAPBPR^HiFi^), which showed drastic enhancement of HLA binding and peptide editing functions (27, 31). Focusing on tapasin, we have performed two complementary DMS screens on the binding interface with HLA-A∗02:01. Using data from both screens, we identified functionally enhanced variants, tapasin-YTY and tapasin-I2. Leveraging monoallelic tapasin knockout (KO) cells, we find that our variants also show robust upregulation of function for the common HLA-A∗01 and HLA-A∗03 allotypes. In parallel assays, we observe enhanced loading of exogenous peptides on HLA-A∗24:02 upon blockade of the TAP transporter (32), when tapasin variants are expressed directly on the plasma membrane. These results provide a general approach for fine-tuning chaperone interactions with diverse HLA allotypes that are useful for potential applications in T cell screening and druggable peptide target discovery.

## Results

### Complementary deep mutational scanning screens reveal a conserved landscape of MHC-I interaction surfaces on tapasin

**Figure 1:**
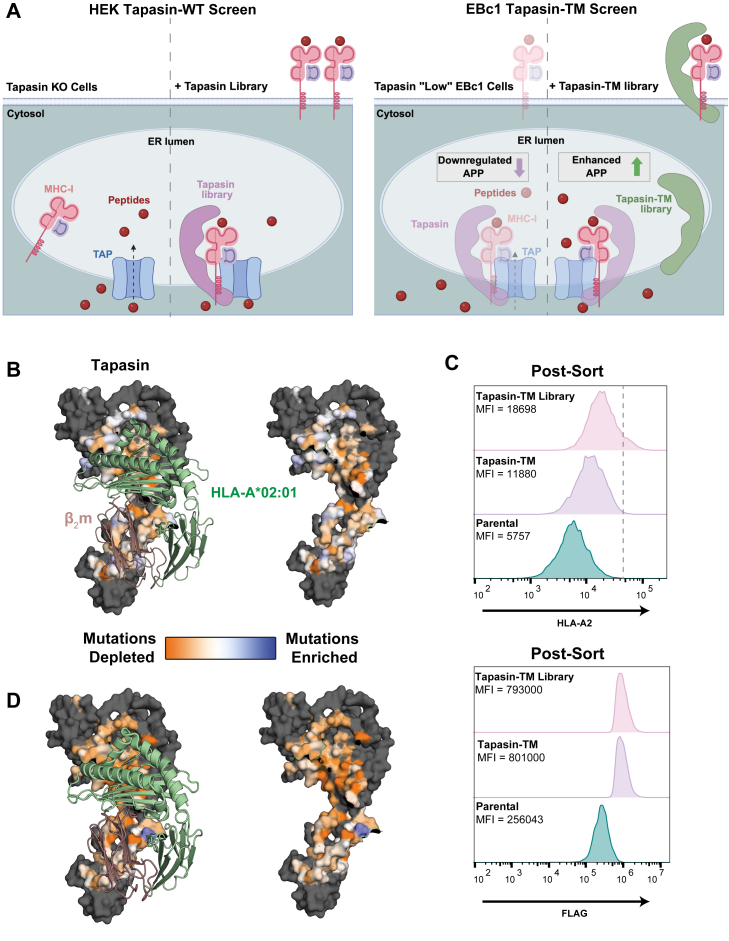
**Deep mutational scanning of tapasin reveals a largely conserved HLA binding interface.***A*, schematic illustrating the tapasin-WT (*left*) and tapasin-TM (*right*) screens. (*left*) Tapasin-KO HEK cells were transduced with WT tapasin library, rescuing surface HLA-A2 expression. (*right*) EBc1 HLA “low” cells were transduced with tapasin-TM, enhancing surface HLA-A2 expression. Created in BioRender. Erdman, M. (2026) https://BioRender.com/zkn7a0k. *B*, Conservation scores from the deep mutational scan of tapasin-WT are mapped onto the surface of tapasin (PDB ID 6ENY) in complex with HLA-A2 (PDB ID 1DUZ). Residues that are highly conserved are shown in *orange*. Sites which are more mutationally tolerant and may contain functional amino acid substitutions are shown in *blue*. Unmutated residues are colored *light gray*. *C*, expression of HLA-A2 and tapasin (measured *via* FLAG staining) after sorting EBc-1 cells for HLA-A2 expression. *D*, conservation scores from the deep mutational scan of tapasin-TM are mapped onto the surface of tapasin in complex with HLA-A2 as shown in B.

To understand how variations in the sequence of tapasin influence interactions with HLA-A∗02:01 (HLA-A2), we analyzed sequencing data from our previously performed DMS screen using a transiently expressed library of wildtype tapasin (tapasin-WT screen) (26). For this screen, HEK tapasin KO cells were transfected with a tapasin library containing 2160 single amino acid substitutions across 108 residues at the HLA binding interface and control regions (Fig. 1*A*). Transfected cells with the highest surface HLA-A2 expression were sorted *via* fluorescence-activated cell sorting (FACS) and deep sequenced (26). Using this data, we calculated the conservation score at each residue. Mapping these values onto a model of the tapasin/HLA-A2 complex reveals a largely conserved mutational landscape where most residues at the HLA-I binding interface are intolerant of mutations (Figs. 1*B* and S1, *A* and *B*). Two regions show an increased mutational tolerance: the V10-K20 loop and the region just behind this loop, which is poised to interact with the tip of HLA-I α_1_ and α_2-1_ helices.

In addition to the tapasin-WT screen, we performed a complementary DMS screen on tapasin-TM, a modified form of tapasin (23). This construct, which replaces the transmembrane (TM) domain of tapasin with that of HLA-G, ablates an ER retention motif that enables tapasin to express throughout the secretory pathway and directly at the cell surface. Analogous to our previous DMS studies focusing on the tapasin homolog TAPBPR (27, 28, 30, 33), this allowed us to examine tapasin’s function beyond the peptide loading complex, providing another window into tapasin/HLA-A2 interactions (Fig. 1*A*) (10). To examine HLA-I/chaperone interactions in an immunologically “cold” background, we transduced NB-EBc1 (EBc1) cells, an HLA-A2+ neuroblastoma cell line with low expression of endogenous tapasin and other APP components, resulting in an extreme case of low HLA-I presentation (23). For this reason, these cells are a biologically relevant system to study tapasin/HLA-I interactions.

For our tapasin-TM screen, cells were transduced with a lentiviral library carrying the same 2160 mutations as done previously for our tapasin-WT library, and sorted for high HLA-A2 expression using the HLA-A2-specific antibody BB7.2 (Fig. S2). Notably, cells sorted for high HLA expression had uniform levels of tapasin, monitored independently using a FLAG surface marker (Fig. 1*C*). Sorted and naïve library populations were deep sequenced, and conservation scores were mapped onto the same model of the tapasin/HLA-A2 complex. The mutational landscape for tapasin-TM is less tolerant than for tapasin-WT, likely due to the different subcellular localizations of each tapasin variant (Figs. 1*D* and S1, *C*–*E*). Tapasin-WT is tethered to the TAP transporter in the ER, where colocalization with other members of the peptide loading complex enhances interactions with HLA-I. This likely reduces the effect of individual mutations on overall function. In contrast, tapasin-TM can express outside of its native compartment and on the cell surface, which amplifies the effect of each mutation and decreases overall mutational tolerance.

### Tapasin variants enhance interactions with HLA-A2

**Figure 2:**
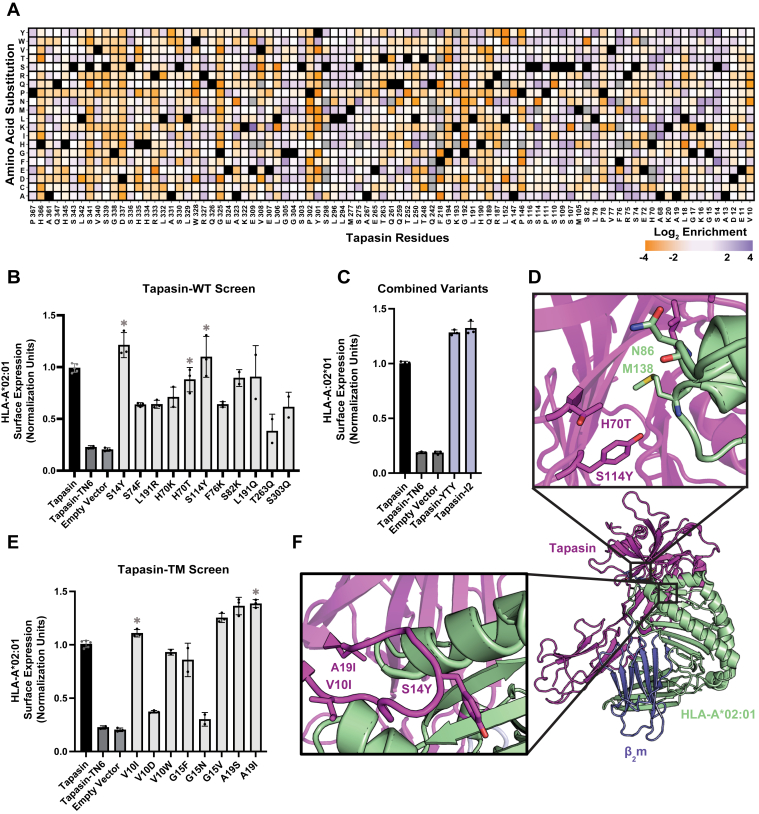
**Mutations identified by two tapasin DMS studies enhance surface HLA-A∗02:01 expression.***A*, HEK Tapasin-KO cells were previously deep mutationally scanned for tapasin-WT mutants that rescued surface HLA-A2 expression. Log_2_ enrichment ratios of each mutation are indicated in *orange* (depleted), *white* (neutral) or *blue* (enriched). Mutations with exceptionally low naïve frequencies (<0.0005) were excluded from analysis and are shown in *gray*. WT residues are shown in *black*. *B-C*, normalized surface expression of HLA-A2 on tapasin KO HEK cells transduced with tapasin variants identified from the (*B*) tapasin-WT screen and (*C*) combined variants. All mutants are approximately 10% transduced. All constructs contain an IRES linked GFP reporter protein and transduction efficiency is reported as the percentage of GFP + cells. Single mutations combined to form combined variants are indicated with an asterisk. *D*, structural model of the tapasin-YTY and variants (*purple*) in complex with HLA-A2 (*green*). Key residues are shown as sticks and colored by element (PDB: 9RCV, 1DUZ). *E*, normalized surface expression of HLA-A2 on tapasin KO HEK cells transduced with tapasin variants identified from the tapasin-TM screen, as shown in *panel B*. *F*, structural model of the tapasin-I2 variant, as shown in panel (*D*). Data presented in panels (*B*, *C*, *D*) are mean ± SD with n = 2 to 5 experimental replicates.

Using the results from the two DMS screens, we wanted to determine whether we could optimize tapasin-HLA interactions by combining promising mutants. From the tapasin-WT screen, we selected eleven highly enriched mutations that appeared to be structurally relevant for enhancing interactions with HLA and assessed their ability to rescue surface HLA-A2 expression in HEK tapasin-KO cells (Fig. 2, *A*, *B*, S3 and S4). Three mutations, S14Y, H70T and S114Y showed an increase in HLA-A2 expression and were combined to form the tapasin-YTY triple mutant (Fig. 2*C*).

These mutations appear to enhance hydrophobic interactions with the tip of the HLA-I α_1_ and α_2-1_ helices proximal to the binding interface. The S14Y mutation lies near the turn of the V10-K20 loop. This loop, and its counterpart in the homologous chaperone TAPBPR, have been shown to be important for tapasin’s peptide-editing function *via* a multitude of mechanisms (10, 12, 16, 17, 19, 26, 27). The S14Y mutation appears to enhance tapasin–HLA interactions *via* hydrophobic packing with the α_1_ and α_2-1_ helices. The S114Y and H70T mutations are set just behind this loop and likely interact with hydrophobic residues at the proximal tip of each helix (Fig. 2*D*).

Enriched mutations in our tapasin-TM screen were largely clustered in three areas: the V10-K20 loop region, adjacent residues H70 and S114, and the IgC region. We also noted that the most enriched mutations tended to be hydrophobic, further indicating that enhanced tapasin/HLA-I interactions are likely driven by hydrophobic packing. Due to the effectiveness of the S14Y mutation in our tapasin-WT screen, as well as the known functional relevance of the V10-K20 loop, we focused on mutations in this region in our tapasin-TM screen (12, 26, 27). We identified eight highly enriched loop mutations and assessed surface HLA-A2 expression in tapasin-KO cells (Figs. 2*E* and S1*E*). Combining two of these mutants, V10I and A19I, we created tapasin-I2. This variant improved HLA expression, likely through enhanced hydrophobic interactions with the tip of the α_1_ and α_2-1_ helices. It is also possible that these mutations stabilize the loop at either anchor point through improved van der Waals interactions with neighboring loop residues (34) (Figs. 2, *C*, *F*, S3 and S4).

### Tapasin-YTY and tapasin-I2 variants show enhanced function across HLA allotypes

**Figure 3:**
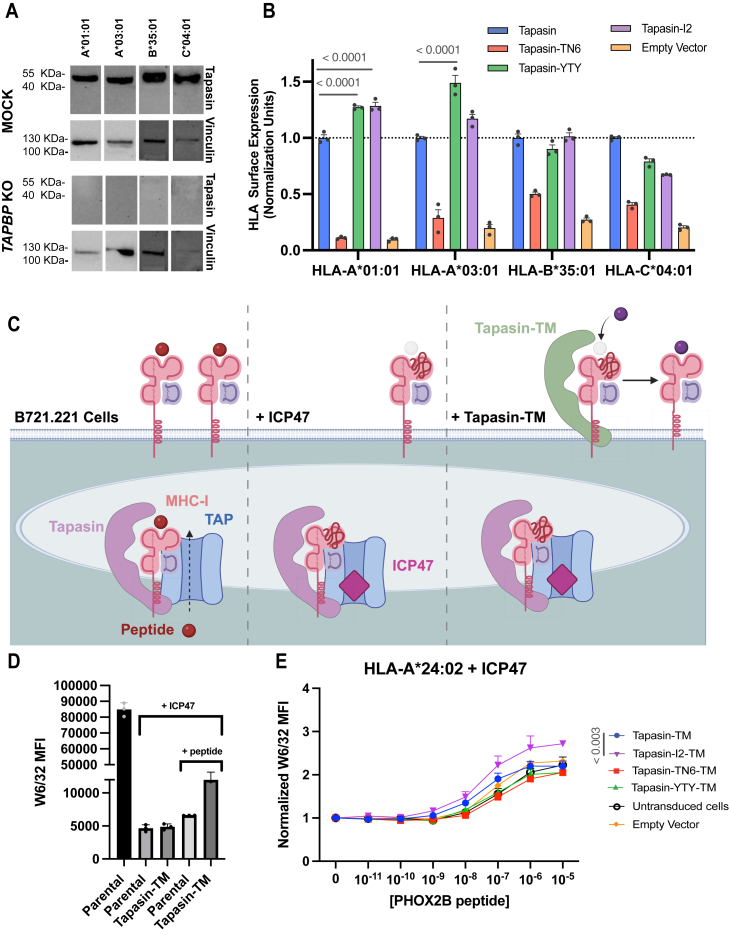
**Tapasin-YTY and Tapasin-I2 variants show enhanced chaperone and editing functions.***A*, CRISPR mediated knock out of *TAPBP* in B721.221 HLA-I monoallelic cell lines. Cells lines were electroporated with Cas9/*TAPBP* sgRNA (tapasin) or nuclease free water. Knock out of *TAPBP* was assessed *via* Western blot staining for tapasin and loading control vinculin. *B*, normalized surface HLA-I expression of B721.221 *TAPBP* KO cells transduced with tapasin variants. A single transduction efficiency is shown for each cell line: HLA-A∗01:01 (40%), HLA-A∗03:01 (10%), HLA-B∗35:01 (15%), and HLA-C∗04:01 (15%). *p* values determined *via* a one-way ANOVA test followed by Sidak’s multiple comparisons test are indicated in *gray*. *C*, inhibition of the TAP transporter (*light blue*) by viral inhibitor ICP47 (*magenta*) decreases the amount of optimally folded HLA molecules at the cell surface. In the presence of exogenous peptide, tapasin-TM facilitates peptide exchange and stabilization of surface HLA molecules, increasing the amount of functional HLA-I at the cell surface. Created in BioRender. Erdman, M. (2026) https://BioRender.com/yeie5r5. *D*, surface expression of HLA-I in parental B721.221 cells and B721.221 cells transduced with ICP47 and Tapasin-TM. Surface HLA-I expression after 2-h incubation with 100 nM PHOX2B peptide is shown under the bracket. *E*, B721.221 HLA-A∗24:02 + ICP47 cells peptide pulsed with PHOX2B. W6/32 MFI is normalized to the no peptide condition. *p* values determined *via* an unpaired *t* test at the highest peptide concentration are indicated in *gray*. Data presented in *panels* (*B*, *D*, *E*) are mean ± SD with n = 3 experimental replicates.

HLA is extremely polymorphic, and different alleles exhibit varying degrees of tapasin dependence for folding and cell surface expression (35, 36). We questioned whether the improved functionality of our tapasin mutants identified using HLA-A2 as a reporter could be maintained for other HLA allotypes. Though our tapasin-YTY and tapasin-I2 variants showed only a modest improvement in HLA-A2 expression relative to their component mutations alone, we reasoned that having multiple mutation sites increased the likelihood that each variant would improve expression across different allotypes. For this reason, we wanted to assess the impact of tapasin-YTY and tapasin-I2 across HLA alleles. To do this, we used B721.221 lymphoblastoid cells, which endogenously lack HLA-I molecules. These cells have been complemented with single HLA-I transgenes, enabling the analysis of HLA molecules on a single-allele basis (37). We knocked out endogenous tapasin (*TAPBP*) using CRISPR/Cas9 editing and assessed the impact of our tapasin variants on surface HLA expression (Fig. 3, *A* and *B*). Interestingly, beyond HLA-A∗02:01, we found that our tapasin-YTY variant also showed enhanced HLA expression in HLA-A∗01:01 and HLA-A∗03:01 cell lines, relative to the WT tapasin control. The tapasin-I2 variant similarly showed enhanced function in HLA-A∗01:01 cells, though this effect was less pronounced in the HLA-A∗03:01 cell line. As expected, neither mutation enhanced expression of any HLA-B∗ or HLA-C∗ alleles tested, likely due to structural divergence resulting from amino acid polymorphisms present in their interaction surfaces (Figs. 3*B*, S5 and S6).

The ability of each variant to enhance expression for divergent HLA-A∗ allotypes can be explained by their potential binding interactions. Two of the tapasin-YTY mutations appear focused on interactions with portions of the α_1_ and α_2-1_ helices which tend to be relatively oligomorphic. The S114Y mutation seems to facilitate hydrogen bond formation with the backbone amide group of M138, stabilizing the interaction with the α_2-1_ helix in an allele-independent manner. Similarly, the H70T mutation likely interacts with the N86 residue or its associated glycan (Fig. 2*D*) (38). This residue and glycan are highly conserved and necessary for proper HLA assembly (12, 38, 39). Similarly, if the enhanced function of tapasin-I2 is due to intra-loop stabilizing effects at loop anchor points, this effect would be recapitulated across alleles (Fig. 2*F*).

To further understand allele-specific functions of our tapasin variants, we assessed how they impacted peptide loading on a cellular membrane. To do this, we synthesized our variant constructs on a tapasin-TM background, which allowed us to evaluate their impact on HLA peptide loading efficiency directly at the cell surface (23, 28, 33). In parental B721.221 HLA-A∗24:02 monoallelic cells, pulsing with the high-affinity peptide QYNPIRTTF from PHOX2B did not result in changes in surface HLA expression (40). Transducing these cells with ICP47, a viral inhibitor of the TAP transporter, resulted in a pronounced decrease in surface expression of folded HLA-I, due to reduced supply of endogenous, high-affinity ligands in the ER lumen for loading onto nascent HLA-I molecules (Fig. 3, *C* and *D*) (41, 42, 43, 44, 45). In this background of B721.221 HLA-A∗24:02 + ICP47 cells, offering exogenous PHOX2B peptide has a modest upregulation of folded HLA-I expression, which is greatly amplified upon lentiviral transduction with tapasin-TM, likely due to the increased availability of peptide-receptive molecules on the cell surface for loading with the exogenous peptide. Using this system, we find that the tapasin-I2-TM variant improved HLA-A∗24:02 peptide loading efficiency, relative to tapasin-TM (Fig. 3*E*). Considering the loop’s role in peptide editing (12, 15, 17, 19), it is probable that the V10I and A19I mutations aid in stabilizing HLA in a peptide receptive conformation, improving peptide loading efficiency and resulting in the observed enhanced expression of folded HLA-I molecules. These results highlight that our engineered variants can improve both the chaperoning and peptide-editing function of tapasin (46, 47).

## Discussion

Due to its central role in antigen processing and presentation, the dedicated HLA chaperone tapasin is uniquely poised to address the poor immunogenicity which characterizes many cancerous and infected cell types (4, 5, 48). It has previously been shown that TAPBPR, a tapasin homolog, can be optimized to boost HLA expression and amplify peptide exchange efficiency (27, 31). Here, we demonstrate that tapasin can similarly be engineered to enhance interactions across HLA alleles.

Through two deep mutational scanning screens of the tapasin-HLA interface, we have shown that tapasin residues in contact with the tip of the α_1_ and α_2-1_ helices as well as the V10-K20 loop have a higher mutational tolerance compared to other portions of the tapasin-HLA interface (Fig. 1, *B* and *D*). This aligns well with the TAPBPR mutational landscape, which was similarly enriched in mutations surrounding the α_2-1_ helix (27). Mutations in these regions of tapasin not only enhance surface expression of HLA-A∗02:01 (Fig. 2*C*), but also appear to improve interactions between tapasin and HLA-A∗01:01 and HLA-A∗03:01 (Fig. 3*B*), indicating that mutational tolerance in these regions may be necessary to accommodate cross allelic differences in HLA structure. Similarly, mutations near the anchor points of the V10-K20 loop enhanced tapasin’s peptide editing function in HLA-A∗24:02 monoallelic cells (Fig. 3*E*). This result, and the overall prominence of V10-K20 loop mutations across our two screens, further highlights this region’s central role in tapasin’s peptide loading and editing function, in line with previous studies (10, 12, 16, 17, 19, 26, 27). Future investigation should consider this loop as a potential area for further enhancing tapasin function.

While we observed significant enhancement in HLA-A∗ expression, this effect was not recapitulated in HLA-B∗ and HLA-C∗, with several alleles showing decreased surface expression in the presence of our tapasin variants (Figs. 3*B*, S5 and S6). Tapasin/HLA binding is impacted by polymorphic residues at the tapasin interface and across the peptide binding groove, and by allele-specific peptide repertoires (27, 35, 36). Since our original screens were performed by isolating mutants with improved HLA-A∗02:01 expression, we likely selected for tapasin variants which preferentially bind to alleles with similar polymorphisms. Although these results demonstrate the difficulty of engineering a universal chaperone, they also highlight a potential path forward. Future screens focusing on mapping the tapasin mutational landscape for interactions with specific alleles could help to identify mutants which enhance HLA-B∗ and/or HLA-C∗ expression. Another important limitation of this study is the lack of direct tapasin-HLA binding data. Due to the inability to express and purify recombinant tapasin, these results only provide an indirect, though illustrative, readout of tapasin/HLA-I interactions.

Though the highly polymorphic nature of HLA poses a formidable challenge in engineering a broadly applicable tapasin variant, we have demonstrated that tapasin can be optimized to enhance HLA expression and function across four HLA-A∗ allotypes, broadening its applicability to multiple disease-relevant HLA alleles. These alleles span a wide range of reported tapasin dependencies (35), further emphasizing the utility of our tapasin variants as a potential tool to enhance pHLA expression and aid in antigen discovery across patient populations (23).

## Experimental procedures

### Fluorescence activated cell sorting of EBc1 cells

EBc1 cells were transduced to 20% efficiency, such that there was approximately one library mutant per cell. Cells were stained with an anti-DYKDDDDK (FLAG) antibody (Biolegend) at a 1:100 dilution to identify cells with surface FLAG expression. Cells were then sorted for FLAG + *via* fluorescence-activated cell sorting to isolate transduced cells. Following the FLAG sort, cells were expanded. These FLAG-sorted cells are the naïve library. After expanding, cells were stained with an anti-HLA-A2 antibody (Biolegend, BB7.2) at a 1:100 dilution, and the cells with the highest surface HLA-A2 expression (top 5%) were sorted. Sorted and naïve cell populations were harvested, genomic DNA was extracted (Monarch), and the tapasin gene was PCR amplified and isolated (Monarch). Isolated amplicons from each cell population were deep sequenced (Azenta), and enrichment values were calculated between the naïve and sorted cell populations.

### Collection and analysis of sequencing data

Genomic DNA was isolated from sorted and naïve library cells using the Monarch Genomic DNA Purification Kit. Tapasin amplicons were then isolated *via* PCR using the following forward and reverse primers: fwd- CCA GAG GTT GAT TAT CGA TAA GC, rev-GCT TCC CGA GCT CTA TAA AAG AG. In each PCR tube, 1 μg of gDNA template was added to 50 μl of NEBNext Ultra II Q5 Mastermix with 5 μl of each primer. Nuclease-free water was added so the total volume was 100 μL. Reactions were run at 98 °C for 30 s followed by 35 repetitions of 98 °C for 7 s, 64 °C for 24 s, and 72 °C for 90 s. Reactions were then held at 72 °C for 2 min. Each reaction mixture was run on a 0.8% agarose gel at 180V for 1.5 h. Bands containing each tapasin amplicon were excised and DNA was extracted using the Monarch Spin DNA Gel Extraction Kit. Extracted amplicons were sent for Illumina Sequencing by Azenta.

Sequencing analysis was done in Python. Using the forward and reverse raw fastq files provided by Azenta, each read was aligned to the reference tapasin sequence to generate a BAM file. GATK DSM analysis tool was used to compute read counts, and mutation rates were computed for each position relative to naïve counts. Log2 enrichment and depletion ratios were then calculated for each position in the sorted library relative to that of the naïve library. Scripts are included in the GEOX submission. Mutations with reasonable naïve frequency ratios (>0.0005) were considered for further analysis. Additional Materials and Methods are included in the supporting information.

## Data availability

This article contains supporting information. Sequencing data are deposited with NCBI Gene Expression Omnibus (GEO) under accession number GSE311856.

## Supporting information

This article contains supporting information.

## References

[1] Cresswell P. (2019). A personal retrospective on the mechanisms of antigen processing. Immunogenetics.

[2] Blum J.S., Wearsch P.A., Cresswell P. (2013). Pathways of antigen processing. Annu. Rev. Immunol..

[3] Yewdell J.W., Bennink J.R. (1999). Mechanisms of viral interference with MHC class I antigen processing and presentation. Annu Rev Cell Dev Biol.

[4] Dhatchinamoorthy K., Colbert J.D., Rock K.L. (2021). Cancer immune evasion through loss of MHC class I antigen presentation. Front. Immunol..

[5] Reddehase M.J. (2002). Antigens and immunoevasins: opponents in cytomegalovirus immune surveillance. Nat. Rev. Immunol..

[6] Kallingal A., Olszewski M., Maciejewska N., Brankiewicz W., Baginski M. (2023). Cancer immune escape: the role of antigen presentation machinery. J. Cancer Res. Clin. Oncol..

[7] Kennedy P.T., Zannoupa D., Son M.H., Dahal L.N., Woolley J.F. (2023). Neuroblastoma: an ongoing cold front for cancer immunotherapy. J. Immunother. Cancer.

[8] Sgourakis N.G., Natarajan K., Ying J., Vogeli B., Boyd L.F., Margulies D.H. (2014). The structure of mouse cytomegalovirus m04 protein obtained from sparse NMR data reveals a conserved fold of the m02-m06 viral immune modulator family. Structure.

[9] Sgourakis N.G., May N.A., Boyd L.F., Ying J., Bax A., Margulies D.H. (2015). A novel MHC-I surface targeted for binding by the MCMV m06 Immunoevasin revealed by solution NMR. J. Biol. Chem..

[10] Blees A., Januliene D., Hofmann T., Koller N., Schmidt C., Trowitzsch S. (2017). Structure of the human MHC-I peptide-loading complex. Nature.

[11] Grandea A.G., Kaer L.V. (2001). Tapasin: an ER chaperone that controls MHC class I assembly with peptide. Trends Immunol..

[12] Jiang J., Taylor D.K., Kim E.J., Boyd L.F., Ahmad J., Mage M.G. (2022). Structural mechanism of tapasin-mediated MHC-I peptide loading in antigen presentation. Nat. Commun..

[13] Sadasivan B., Lehner P.J., Ortmann B., Spies T., Cresswell P. (1996). Roles for Calreticulin and a novel glycoprotein, tapasin, in the interaction of MHC class I molecules with TAP. Immunity.

[14] Paulsson K.M., Kleijmeer M.J., Griffith J., Jevon M., Chen S., Anderson P.O. (2002). Association of tapasin and COPI provides a mechanism for the retrograde transport of major histocompatibility complex (MHC) class I molecules from the Golgi complex to the endoplasmic reticulum. J. Biol. Chem..

[15] Lan B.H., Becker M., Freund C. (2023). The mode of action of tapasin on major histocompatibility class I (MHC-I) molecules. J. Biol. Chem..

[16] Hafstrand I., Sayitoglu E.C., Apavaloaei A., Josey B.J., Sun R., Han X. (2019). Successive crystal structure snapshots suggest the basis for MHC class I peptide loading and editing by tapasin. Proc. Natl. Acad. Sci. U. S. A..

[17] Fisette O., Schröder G.F., Schäfer L.V. (2020). Atomistic structure and dynamics of the human MHC-I peptide-loading complex. Proc. Natl. Acad. Sci. U. S. A..

[18] Fleischmann G., Fisette O., Thomas C., Wieneke R., Tumulka F., Schneeweiss C. (2015). Mechanistic basis for epitope proofreading in the peptide-loading complex. J. Immunol..

[19] Lan H., Abualrous E.T., Sticht J., Fernandez L.M.A., Werk T., Weise C. (2021). Exchange catalysis by tapasin exploits conserved and allele-specific features of MHC-I molecules. Nat. Commun..

[20] Darabi A., Thuring C., Paulsson K.M. (2014). HLA-I antigen presentation and tapasin influence immune responses against malignant brain tumors-considerations for successful immunotherapy. Anti-Cancer Agents Med. Chem..

[21] Shionoya Y., Kanaseki T., Miyamoto S., Tokita S., Hongo A., Kikuchi Y. (2017). Loss of tapasin in human lung and colon cancer cells and escape from tumor-associated antigen-specific CTL recognition. Oncoimmunology.

[22] Park B., Kim Y., Shin J., Lee S., Cho K., Früh K. (2004). Human cytomegalovirus inhibits tapasin-dependent peptide loading and optimization of the MHC class I peptide cargo for immune evasion. Immunity.

[23] Hwang D., Erdman M.C., Adhikari S., Pantula R., Epstein K., Li P. (2026). HLA-Shuttle: a system for enhancing antigen presentation in immunologically cold tumors. Sci. Adv..

[24] Sokol L., Koelzer V.H., Rau T.T., Karamitopoulou E., Zlobec I., Lugli A. (2015). Loss of tapasin correlates with diminished CD8+ T-cell immunity and prognosis in colorectal cancer. J. Transl. Med..

[25] Boyle L.H., Hermann C., Boname J.M., Porter K.M., Patel P.A., Burr M.L. (2013). Tapasin-related protein TAPBPR is an additional component of the MHC class I presentation pathway. Proc. Natl. Acad. Sci. U. S. A..

[26] McShan A.C., Devlin C.A., Morozov G.I., Overall S.A., Moschidi D., Akella N. (2021). TAPBPR promotes antigen loading on MHC-I molecules using a peptide trap. Nat. Commun..

[27] Sun Y., Papadaki G.F., Devlin C.A., Danon J.N., Young M.C., Winters T.J. (2023). Xeno interactions between MHC-I proteins and molecular chaperones enable ligand exchange on a broad repertoire of HLA allotypes. Sci. Adv..

[28] McShan A.C., Devlin C.A., Overall S.A., Park J., Toor J.S., Moschidi D. (2019). Molecular determinants of chaperone interactions on MHC-I for folding and antigen repertoire selection. Proc. Natl. Acad. Sci. U. S. A..

[29] McShan A.C., Natarajan K., Kumirov V.K., Flores-Solis D., Jiang J., Badstübner M. (2018). Peptide exchange on MHC-I by TAPBPR is driven by a negative allostery release cycle. Nat. Chem. Biol..

[30] McShan A.C., Devlin C.A., Papadaki G.F., Sun Y., Green A.I., Morozov G.I. (2022). TAPBPR employs a ligand-independent docking mechanism to chaperone MR1 molecules. Nat. Chem. Biol..

[31] Sun Y., Pumroy R.A., Mallik L., Chaudhuri A., Wang C., Hwang D. (2025). CryoEM structure of an MHC-I/TAPBPR peptide-bound intermediate reveals the mechanism of antigen proofreading. Proc. Natl. Acad. Sci. U. S. A..

[32] Grossmann N., Vakkasoglu A.S., Hulpke S., Abele R., Gaudet R., Tampé R. (2014). Mechanistic determinants of the directionality and energetics of active export by a heterodimeric ABC transporter. Nat. Commun..

[33] Ilca F.T., Neerincx A., Wills M.R., de la Roche M., Boyle L.H. (2018). Utilizing TAPBPR to promote exogenous peptide loading onto cell surface MHC I molecules. Proc. Natl. Acad. Sci. U. S. A..

[34] Stolz M., Sušac L., Fahim A., Keller R., Saggau L., Mancia F. (2026). Architectural principles of transporter-chaperone coupling within the native MHC I peptide-loading complex. Sci. Adv..

[35] Bashirova A.A., Viard M., Naranbhai V., Grifoni A., Garcia-Beltran W., Akdag M. (2020). HLA tapasin independence: broader peptide repertoire and HIV control. Proc. Natl. Acad. Sci. U. S. A..

[36] Rizvi S.M., Salam N., Geng J., Qi Y., Bream J.H., Duggal P. (2014). Distinct assembly profiles of HLA-B molecules. J Immunol..

[37] Abelin J.G., Keskin D.B., Sarkizova S., Hartigan C.R., Zhang W., Sidney J. (2017). Mass spectrometry profiling of HLA-Associated peptidomes in mono-allelic cells enables more accurate epitope prediction. Immunity.

[38] Domnick A., Winter C., Sušac L., Hennecke L., Hensen M., Zitzmann N. (2022). Molecular basis of MHC I quality control in the peptide loading complex. Nat. Commun..

[39] van Hateren A., Elliott T. (2023). Visualising tapasin- and TAPBPR-assisted editing of major histocompatibility complex class-I immunopeptidomes. Curr. Opin. Immunol..

[40] Sun Y., Florio T.J., Gupta S., Young M.C., Marshall Q.F., Garfinkle S.E. (2023). Structural principles of peptide-centric chimeric antigen receptor recognition guide therapeutic expansion. Sci. Immunol..

[41] Goldsmith K., Chen W., Johnson D.C., Hendricks R.L. (1998). Infected Cell Protein (ICP)47 enhances Herpes Simplex virus neurovirulence by blocking the CD8+ T cell response. J. Exp. Med..

[42] Radosevich T.J., Seregina T., Link C.J. (2003). Effective suppression of class I major histocompatibility complex expression by the US11 or ICP47 genes can be limited by cell type or interferon-gamma exposure. Hum. Gene Ther..

[43] Tomazin R., van Schoot N.E.G., Goldsmith K., Jugovic P., Sempé P., Früh K. (1998). Herpes Simplex virus type 2 ICP47 inhibits human TAP but not mouse TAP. J. Virol..

[44] Sim M.J.W., Lu J., Spencer M., Hopkins F., Tran E., Rosenberg S.A. (2020). High-affinity oligoclonal TCRs define effective adoptive T cell therapy targeting mutant KRAS-G12D. Proc. Natl. Acad. Sci. U. S. A..

[45] Sim M.J.W., Malaker S.A., Khan A., Stowell J.M., Shabanowitz J., Peterson M.E. (2017). Canonical and cross-reactive binding of NK cell inhibitory receptors to HLA-C allotypes is dictated by peptides bound to HLA-C. Front Immunol..

[46] Fisette O., Wingbermühle S., Tampé R., Schäfer L.V. (2016). Molecular mechanism of peptide editing in the tapasin–MHC I complex. Sci. Rep..

[47] Boulanger D.S.M., Douglas L.R., Duriez P.J., Kang Y., Dalchau N., James E. (2022). Tapasin-mediated editing of the MHC I immunopeptidome is epitope specific and dependent on peptide off-rate, abundance, and level of tapasin expression. Front Immunol..

[48] Wang L., Geng H., Liu Y., Liu L., Chen Y., Wu F. (2023). Hot and cold tumors: immunological features and the therapeutic strategies. MedComm (2020).

