# Supplementary material for: Engineering high-fidelity tapasin variants to enhance MHC-I antigen presentation
Source: J Biol Chem. 2026 Mar 21;302(5):111400. doi: 10.1016/j.jbc.2026.111400 (PMC13125989; doi:10.1016/j.jbc.2026.111400)
Supplement: Supplementary Figures [file mmc1.pdf]

Engineering high-fidelity tapasin variants to enhance MHC-I antigen presentation

Molly C. Erdman, Kaya Epstein, Daniel Hwang, Radia M. M. Khan, Shirley M. Sun, Nikolaos G. Sgourakis

This PDF file includes:

Extended Materials and Methods

Figs S1 to S6

Extended Materials and Methods

Cell Lines

Expi293F (Thermo Fisher) cells were cultured in Expi293 media (Thermo Fisher) supplemented with 1% Penicillin Streptavidin (Thermo Fisher) at 37 °C, 125RPM and 8% CO2. *TAPBP*-KO cells were previously generated (24).

B721.221 and EBc1 cells were cultured in Advanced RPMI Medium (Thermo Fisher) supplemented with 10% premium heat inactivated fetal bovine serum, 5% HEPES, 5% Glutamine, 5% Penicillin Streptavidin (Thermo Fisher). Cells were cultured at 37 °C and 5% CO2. B721.221 cells were obtained from Derin Keskin at the Dana-Farber Cancer Institute. EBc1 cells were obtained from John Maris at the Children's Hospital of Philadelphia.

All cells tested negative for mycoplasma.

Lentiviral Production and Transduction

To produce lentiviruses, Lenti-X 293T cells (Takara Bio) were transfected with a pSFFV or CMV transfer vector containing the gene of interest, the psPAX2 packaging vector, and the pMD2.G envelop vector. Transductions were performed by adding concentrated lentivirus to the desired cells. For pSFFV lentiviruses, transduction efficiency was determined via intracellular staining with a fluorophore conjugated anti-DYKDDDDK (FLAG) antibody (L5, Biolegend). For CMV lentiviruses, transduction efficiency was measured via GFP fluorescence.

CRISPR-Cas9 Editing

721.221 monoallelic cells were edited by electroporation of CRISPR-Cas9 RNPs. Cas9 was complexed with *TAPBP* specific sgRNA (5'-GAACCAACACUCGAUCACCG-3') at a 1:3 ratio (Synthego/Editco). Cells were then electroporated using a Lonza 4D-Nucleofector® X Unit program EH-100 and buffers from p3 primary cell 4d-nucleofector® x kit (Lonza). RNPs were assembled in the following order in 16 well strips: 5µl P3 Buffer, 1.2 µL sgRNA (100 µM), 2 µL Cas9 (20 µM) per reaction. 8.2 µL of this mixture was combined with 200,000 cells in 15 µL P3 buffer.

Knock out of *TAPBP* was assessed via Western Blot. 500,000 cells were lysed in 50 µL of digitonin solution (1% digitonin in 20mM Tris-HCl 150mM NaCl pH 7.5) containing Halt protease inhibitor cocktail (Thermo Scientific) for 30 minutes at 4C with gentle agitation. Cell solutions were then centrifuged for 30 minutes at 12,000g. Cell lysate was reduced by adding

7 µL of LDS sample buffer containing Sample Reducing Agent (Invitrogen) and boiled at 97C for 5 minutes. 20 µL of reduced lysate was loaded onto a 4-12% Bis-Tris gel (Invitrogen) and run for 35 minutes at 200V. Gels were transferred to nitrocellulose membranes (Biorad) according to the XCell II Blot Module manual (Invitrogen). Membranes were blocked in TBS-T buffer (25mM Tris, 150mM sodium chloride, 0.05% Tween-20) with 5% milk powder for one hour with gentle agitation. Following blocking, membranes were stained in the same 5% milk TBS-T buffer with 0.5 µg/mL of anti-tapasin (7F6, Millipore) or anti-vinculin (W18245A, Biolegend) antibodies overnight at 4C with slight agitation. The following day, membranes were washed in TBS-T buffer 6 times for 5 minutes each with slight agitation. Membranes were then stained with 1:5,000 dilution of HRP-conjugated anti-rat IgG antibody (Thermo Scientific) in 5% milk TBS-T buffer for 1 hour with slight agitation. Membranes were washed again as described above, and visualized using the Pierce ECL Western kit (Thermo Scientific). Antibody specificity was confirmed through use of a control *TAPBP* KO cell line and a cell line with endogenous tapasin.

Assessing Surface HLA Expression via Flow Cytometry

To assess surface HLA expression, 50,000 cells were plated in a 96 well plate. Cells were stained with Live/Dead NearIR cell stain (Invitrogen) at a 1:1000 dilution for 15 minutes at room temperature. Cells were then stained with PE or APC-BB7.2 or APC-W6/32 (Biolegend) at a 1:100 dilution for 30 minutes on ice. Excess antibody was removed, and cells were fixed by resuspending each well in 50uL of Fix/Perm Medium A (Thermo Fisher) for 15 minutes at room temperature. Cells were then washed and resuspended in 100uL of FACS buffer (2.5% FBS, 0.02% sodium azide, 10% PBS) and surface HLA-A2 or surface HLA-ABC expression was assessed via PE or APC MFI. Since tapasin constructs used in Figures 2 and 3 contain an IRES linked GFP, transduction efficiency was assessed by calculating the percentage of GFP positive cells. The BB7.2 of W6/32 MFI of GFP- cells was subtracted from that of the GFP+ cells to account for background staining differences. To calculate normalization units, each sample was normalized to the tapasin-WT transduced samples. Statistical significance was determined via a one way Anova test followed by Sidak's multiple comparisons test.

Peptide Pulse Assay with ICP47-Transduced B721.221 Cells

B721.221 cells expressing a single HLA class 1 allele were transduced with ICP47 such that greater than 90% of cells were transduced. ICP47 constructs contain mCherry, and transduction efficiency was assessed by measuring mCherry expression via flow cytometry.

To assess the impact of local peptide concentration of surface HLA expression, 100,000 B721.221-ICP47 cells were plated in 96 well plates in 50uL of serum-free RPMI media (Thermo Fisher). Lyophilized peptide (Genscript) was resuspended in DMSO and diluted to 200uM. Starting at 200uM, peptides were serially diluted in serum-free media to create 7-11 10-fold dilutions. 50uL of each peptide dilution was added to the correct well containing B721.221-ICP47 cells. Plates were gently mixed using a plate shaker (800RPM, 30 seconds) and incubated at 37C for 2 hours. Following the incubation period, cells were stained, and HLA expression was assessed as described in “Assessing Surface HLA Expression via Flow Cytometry”.

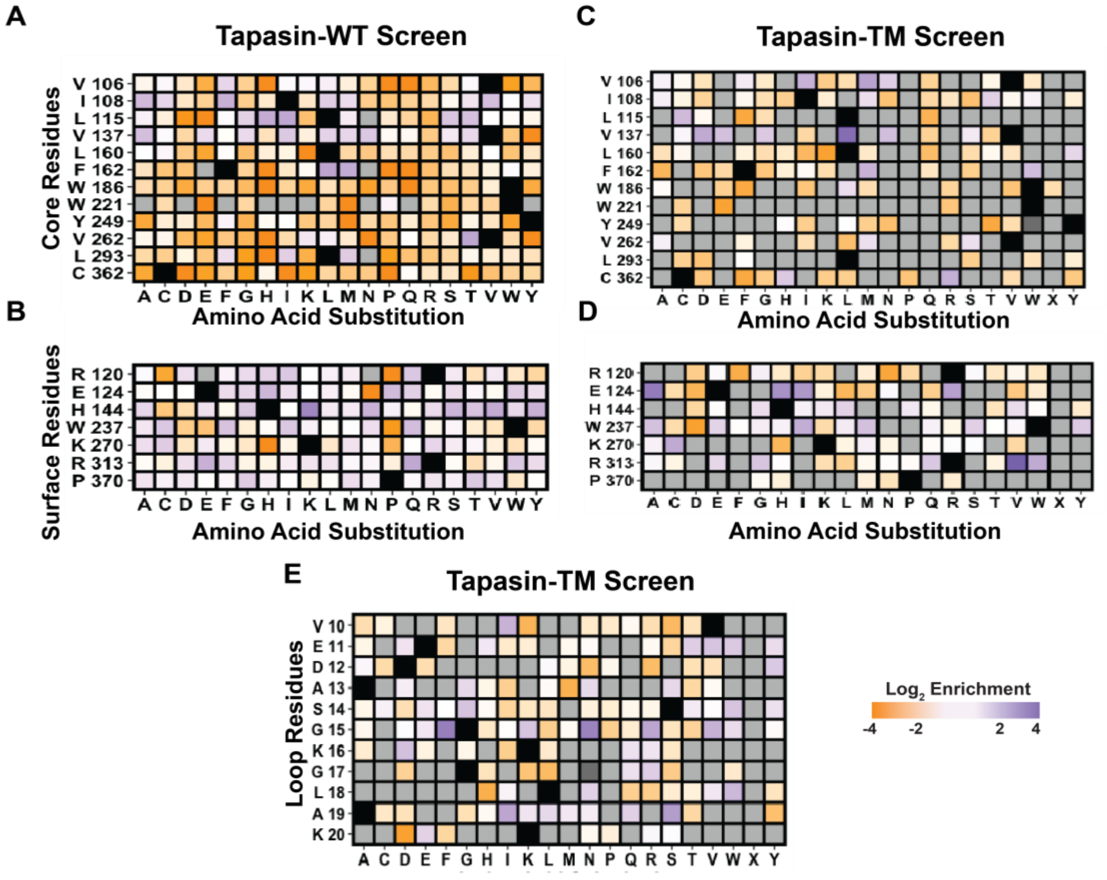

Supplementary Figure 1: Plotted Log_2 enrichment values for control and experimental residues from the tapasin-WT and tapasin-TM DMS screens. Enriched mutations are shown in blue, depleted mutations are shown in orange, and wildtype residues are indicated in black. Mutations with exceptionally low frequencies in the naïve library (<0.0005) were excluded from analysis and are indicated in grey. **(A-B)** Control residues from the tapasin-WT screen at the **(A)** core and **(B)** surface of tapasin. **(C-D)** Control **(C)** core and **(D)** surface residues from the tapasin-TM screen. **(E)** Experimental residues in the loop region of the tapasin-TM screen.

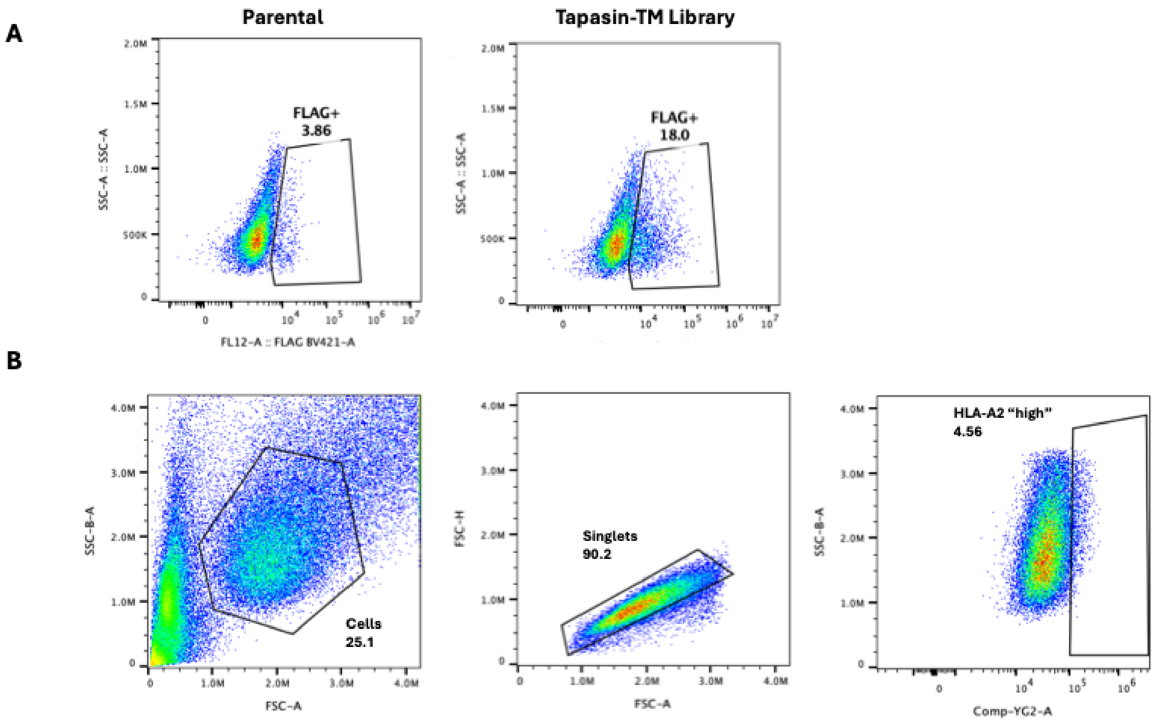

Supplementary Figure 2: Gating strategies for fluorescence-activated cell sorting. **(A)** EBc-1 cells transduced with the Tapasin-TM library were first sorted to isolate cells transduced with the library, which contains a FLAG tag (DYKDDDDK). **(B)** Following sorting and expansion of the naïve library, these cells were sorted to identify those with the highest surface HLA-A2 expression. Three sequential gates, indicted by black polygons, were used to identify EBc-1 cells with the highest surface HLA-A2 expression. Cells were first gated by FSC-A/SSC-A to distinguish the main cell population from debris. Next, this cell population was gated on FSC-H/FSC-A to exclude any doublet or triplet cells from analysis. Finally, the singlet cell population was gated on SSC-A/PE in order to isolate cells with the highest surface HLA-A2 expression (top 5%). All sorting was done on a BD FACSAria™ Fusion Flow Cytometer.

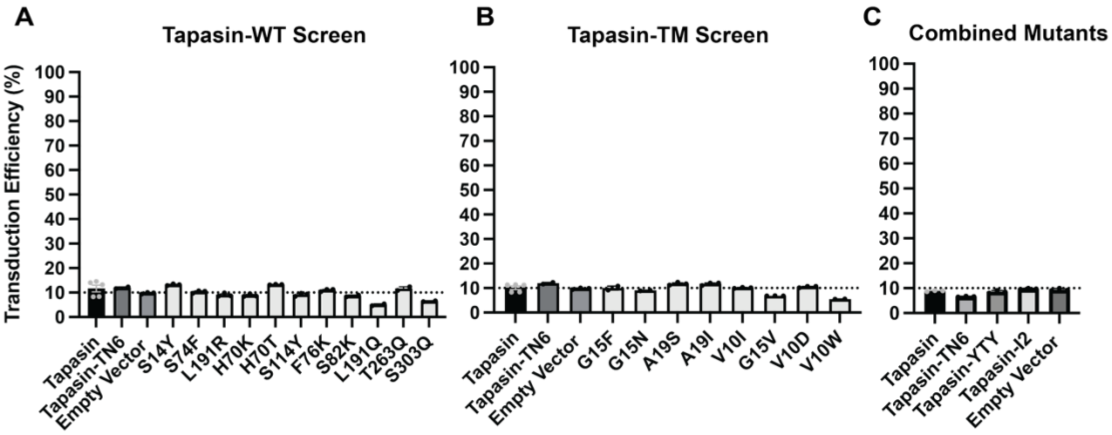

Supplementary Figure 3: Transduction efficiencies of tapasin constructs expressed in HEK tapasin-KO cells. HEK tapasin-KO cells were transduced with tapasin constructs from the **(A)** tapasin-WT and **(B)** tapasin-TM screens, and **(C)** the combined variants. Constructs contain an IRES linked GFP and transduction efficiency was calculated by the percentage of GFP+ cells. At transduction efficiencies of about 10%, nearly all cells contain only one integration event. Data presented are mean ± SD with n = 2-5 experimental replicates.

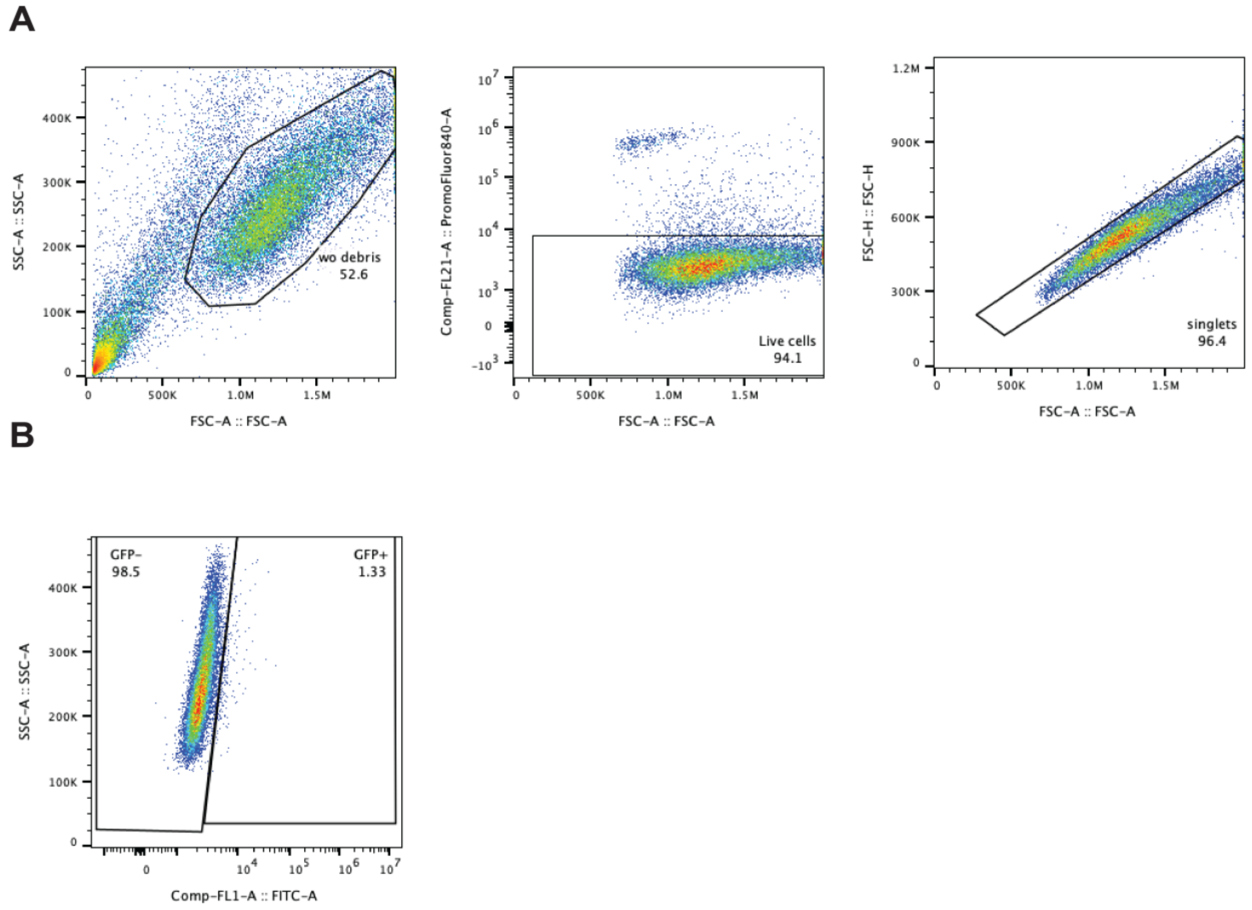

Supplementary Figure 4: Representative Gating Strategy for Flow Cytometry Assays. (A) Cells were first gated by FSC-A/SSC-A to distinguish the main cell population from debris. Next, cells were gated to isolate live cells. Next, this cell population was gated on FSC-H/FSC-A to exclude any doublet or triplet cells from analysis. **(B)** Finally, the singlet cell population was gated on SSC-A/GFP in order to isolate GFP+ cells, using untransduced cells as a gating control (shown).

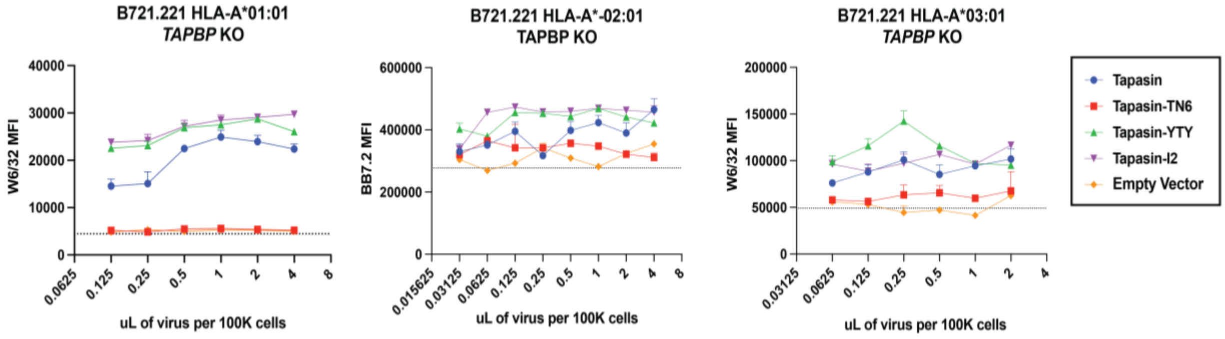

Supplementary Figure 5: Surface HLA-I expression in B721.221 HLA-A* *TAPBP* KO cells transduced with tapasin variants. HLA-I MFI of Parental B721.221 tapasin-KO cells is represented by a dotted line. Data presented are mean ± SD with n = 3 experimental replicates.

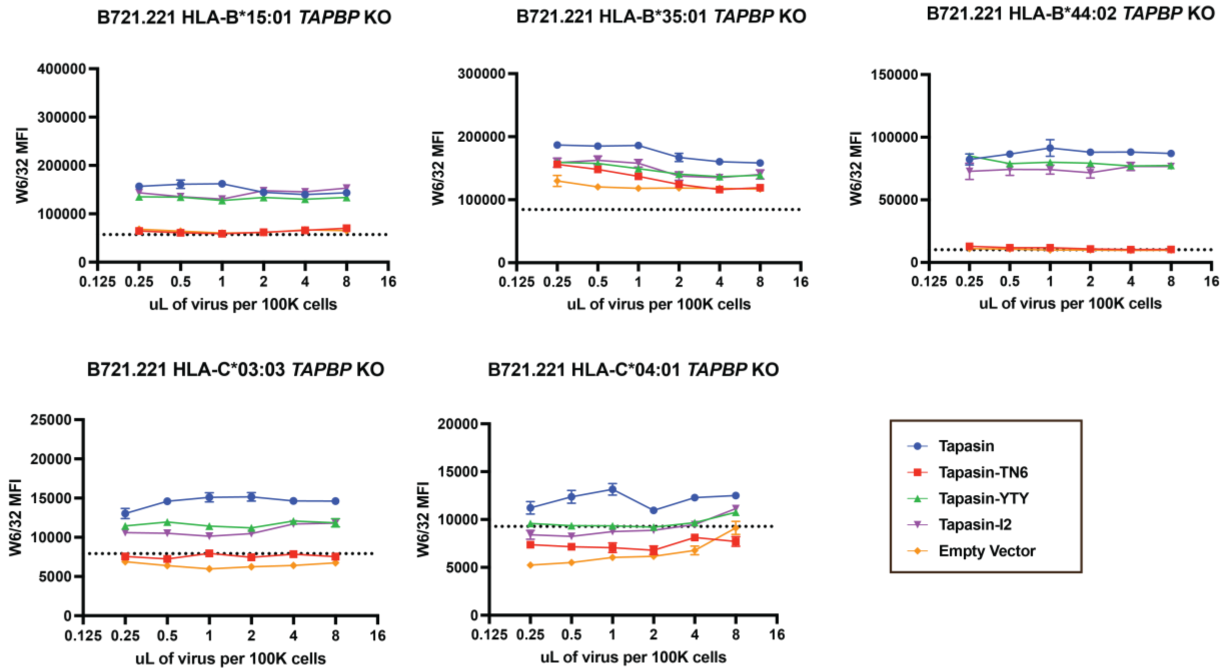

Supplementary Figure 6: Surface HLA-I expression in B721.221 HLA-B* and HLA-C* *TAPBP* KO cells transduced with tapasin variants. HLA-I MFI of Parental B721.221 tapasin-KO cells is represented by a dotted line. Data presented are mean ± SD with n = 3 experimental replicates.
